# Occupational health for an ageing workforce: do we need a geriatric perspective?

**DOI:** 10.1186/1745-6673-1-8

**Published:** 2006-05-23

**Authors:** Gerald Choon-Huat Koh, David Koh

**Affiliations:** 1 Community, Occupational and Family Medicine Department, Yong Loo Lin School of Medicine, MD3, 16 Medical Drive, 117597, Singapore

## Abstract

Extending retirement ages and anti-age discrimination policies will increase the numbers of older workers in the future. Occupational health physicians may have to draw upon the principles and experience of geriatric medicine to manage these older workers. Examples of common geriatric syndromes that will have an impact on occupational health are mild cognitive impairment and falls at the workplace. Shifts in paradigms and further research into the occupational health problems of an ageing workforce will be needed.

## Introduction – the ageing workforce

The world is undergoing unprecedented ageing and in many developed countries, the workforce is contracting due to falling birthrates, longer life expectancies and changing population demographics [1]. Experts have warned that if society continues to reduce the number of people over the age of 50 who are not actively working, economies will suffer a cumulative annual loss of GDP [2]. Some countries like the UK are already introducing anti-age discrimination policies laws and retirement ages are projected to increase in the coming years [3]. Employers now have to face the prospect of having workers in their sixties. In New Zealand, the number of older persons aged 45 to 65 years is expected to increase from 35% to 45% within the working-age population between 2001 and 2051 [4]. The International Labour Organisation estimates that the number of economically active persons aged 65 years and above will increase from 83.2 million persons in the world in 2000 to 136 million persons by 2020 [5]. Occupational physicians are accustomed to managing middle-aged workers and their associated health problems but are we ready to manage elderly-related illnesses that may impact worker performance and health?

## What does geriatric medicine has to offer?

Geriatrics is the branch of medicine that is devoted to the care of older people [6]. The relatively young discipline addresses the unique needs and circumstances of the elderly and is characterized by recognition of geriatric syndromes. Examples of conditions that affect the elderly include falls, impaired cognition, disability, malnutrition, incontinence and iatrogenesis. At first glance, most of these syndromes are associated with advanced age and it is unlikely that such an old person would still be working and hence be seen by an occupational physician. However, when one considers that many geriatric syndromes can present in fifth decade of life, it becomes apparent that knowledge of geriatric syndromes may be relevant to occupational health. This paper will use 2 common geriatric syndromes that may impact on the occupational health of older workers to illustrate this.

## Dementia and mild cognitive impairment

Dementia is often thought of as a psychiatric disease of the old. However, a paper by McMurtray et al found that 30% of patients presenting at the Veteran's Affairs Medical Center Memory Disorders clinic between 2001 and 2004 for evaluation of memory or cognitive decline had an age of onset of less than 65 years (early onset dementia [EOD]) [7]. Compared to the late-onset dementia [LOD] group, the EOD patients were less severely impaired on presentation. Hence, it is possible that an older worker may present with onset of dementia before retirement which can interfere with work or endanger the lives of fellow co-workers. It is interesting to note that the EOD group had significantly more dementia attributed to traumatic brain injury, alcohol abuse, human immunodeficiency virus (HIV) and frontotemporal lobe degeneration than the LOD patients which had significantly more Alzheimer's disease compared to the EOD group. With the exception of the last condition, the causes of EOD are largely preventable. Hence, occupational physicians can play an important role in the prevention, early detection and treatment of EOD.

One of the earliest cognitive domains lost in dementia is executive functioning involving understanding complex material, and this can occur before memory loss [8]. This has implications because most clinical diagnostic criteria for dementia involve subjective and objective memory impairment and functional decline. Even the clinical diagnostic criteria for mild cognitive impairment (MCI) requires subjective or objective memory loss but without functional impairment (Table 1) [9]. An older worker in a job requiring high-level mental functioning may be making poor decisions and losing millions of dollars for the company long before anyone perceives any impairment of memory. Clinically, the distinction between benign senescent forgetfulness (normal process of ageing) and mild cognitive impairment is subtle and this makes the detection of early loss of executive functioning extremely difficult to detect.

**Table 1:** Various definitions of mild cognitive impairment (Adapted from Chong and Sahadevan [9])

	**Amnestic MCI**	**AACD**	**AAMI**	**CIND**	**CDR = 0.5**
**Subjective memory impairment**	+	+	+	NR	+
**Subjective non-memory impairment**	-	NR	NR	NR	NR
**Objective memory impairment**	+ ^a^	+ ^b^	+ ^c^	+	+
**Objective non-memory impairment**	-	NR	NR	NR	NR
**Functional decline**	NR	NR	NR	NR	+/-
**Functional impairment**	-	NR	NR	NR	-

Abbreviations MCI = mild cognitive impairment; AACD = age-associated cognitive decline; AAMI = age-associated memory impairment, CIND = cognitive impairment no dementia; CDR = clinical dementia rating scale; the score of 0.5 is used to denote, MCI + = must be present for diagnosis; - = must be absent for diagnosis; +/- = may or may not be present for diagnosis; NR = not required (or not mentioned as criteria for diagnosis); a: >1.5 SD below age-matched controls; b: within normal limits given person's age; c: >1 SD below mean for young adults.

Fitness for work for workers which require intact cognition will continue to be a challenge with older workers. The earliest an occupational health physician can hope to detect cognitive decline would be when a worker has MCI. This intermediate stage between normal ageing and dementia has received increasing attention because current therapies for dementia are most effective at the early stages and 12% of cases with MCI convert to dementia annually, reaching 80% at 6 years follow-up [10]. Unfortunately, there is currently no consensus guideline for the diagnosis of mild cognitive impairment but there is evidence for its continued monitoring and treatment [11]. Current cognitive screening tools to detect dementia have not been validated to detect MCI and clinicians have to rely on special cognitive tests. Prospective studies of people with memory-loss MCI have shown that tests involving episodic memory (such as delayed recall of word lists [12] and associative learning [13]), semantic memory [14], attention processing [15] and mental speed can consistently predict which patients will develop dementia. Conversely, in a retrospective study of people with MCI who later developed Alzheimer's dementia, verbal and visual memory, associative learning, vocabulary, executive function and other verbal tests of general intelligence were impaired at baseline [16]. Such tests should be administered by trained personnel and occupational physicians may need training in such assessments.

## Falls and injuries at the workplace

Falls and injuries are common in the workplace but for older persons, they are associated with greater morbidity and mortality [17,18]. Slips, trips and falls are more common among older workers [19] and the resulting occupational injuries are more likely to result in hospitalization [20], fatalities [21] and fractures, particularly among older women [22]. However, falls in older persons are different from the younger population because there is a higher prevalence of medical problems that predispose older persons to falls. Examples of such medical problems that increase the risk of falls and injuries include strokes, dementia, cataracts, age-related macular degeneration, Stokes-Adam attacks from cardiac arrhythmias, vertebro-basilar insufficiency from cervical spondylosis, anaemia, medications with anti-cholinergic properties (e.g. anti-histamines, tricyclic anti-depressants) and postural hypotension from anti-hypertensives or dehydration.

When an older worker falls often, there is a need to move beyond treating injuries and improving workplace safety and towards a thorough assessment of the older worker to ascertain why a previously well worker is now sustaining falls and injuries at the workplace. There have been few published studies on the assessment of risk factors for falls among older workers at the workplace. Evidence from e geriatric medicine literature has consistently shown that multi-factorial assessment for falls risk factors, followed by interventions targeted at identified risk factors, have been effective in preventing further falls [23-25]. Such targeted assessment and management strategies have been found by a Cochrane Database Systematic Review to reduce occurrence of falls among older persons in the community by 25 to 39% [26]. Specific recommendations for fall risk factor assessment are summarized in Table 2.

**Table 2:** Recommended Components of a Clinical Assessment and Management of Older Persons with Previous Falls (Adapted from Tinetti [27])

**Risk Factor**	**Management**
Circumstances of previous falls	Changes in environment to reduce the likelihood of recurrent falls.
Medication use - High risk medications (e.g. benzodiazepines, sedatives, neuroleptics, anti-depressants, anti-convulsants, Class 1A anti-arrhythmics) - Polypharmacy (4 or more medications)	Review and reduction of medications
Vision - Acuity <20/60 - Decreased depth perception - Decreased contrast sensitivity - Cataracts	- Ample lighting - Avoidance of multifocal glasses while walking - Referral to ophthalmologist
Postural blood pressure (after 5 mins in a supine position, immediately after standing and 2 mins after standing) - >20 mmHg or (>20%) drop in systolic pressure, with or without symptoms, either immediately or after 2 min of standing, is significant	Diagnosis and treatment of underlying cause, if possible. Review and reduction of medications; modification of salt restriction, adequate hydration, pressure stockings; fludrocortisone therapy if above strategies fail
Balance and gait - Patient's report or observed unsteadiness. - Impairment on brief assessment (e.g. Get-Up-And-Go test)	Diagnosis and treatment of underlying cause, if possible. Review and reduction of medications; referral to physical therapist for assistive devices and gait, balance and strength training
Targeted neurological examination - Impaired proprioception - Impaired cognition - Decreased muscle strength	Diagnosis and treatment of underlying cause, if possible; increase proprioceptive input (e.g. with assistive device or appropriate footwear that encases the foot and has a low heel and thin sole); review and reduction of medications; referral to physical therapist for assistive devices and gait, balance and strength training
Targeted musculoskeletal examination - examination of legs - examination of feet	Diagnosis and treatment of underlying cause, if possible; referral to physical therapist for assistive devices and gait, balance and strength training; use appropriate footwear, referral to podiatrist
Targeted cardiovascular examination - Syncope - Arrhythmia	Diagnosis and treatment of underlying cause, if possible; referral to cardiologist

To date, there is no randomized control trial to determine effectiveness of interventional strategies to reduce the occurrence of falls among older persons in the workplace, so occupational physicians may need to turn to past studies on older persons in the community. Successful interventions to reduce falls include review and possible reduction of medications, balance and gait training, muscle-strengthening exercises, evaluation and strategies to reduce postural hypotension and targeted cardiovascular assessment and treatment. (Table 2)

The role of laboratory testing and other investigations in fall assessment has not been well studied [27]. Laboratory tests that may be reasonable in the assessment of an older worker who has fallen include a complete blood count (to detect anaemia or a raised total white count suggesting a sub-clinical infection), serum electrolytes, glucose, vitamin B12, blood urea nitrogen and creatinine (to detect serum abnormalities that can cause impaired judgement or muscle weakness) and thyroid function (to detect hypothyroidism which can cause confusion and muscle weakness). In occupational settings with exposure to neurotoxins that can cause cognitive impairment, neuropathy and muscle weakness, such as metals (e.g. arsenic, lead, manganese), solvents (e.g. carbon disulphide, n-hexane and methyl-n-butyl ketones) and pesticides (e.g. organochlorine and organophosphate compounds), screening for these chemicals would be vital. Neuro-imaging is only needed when there is history of head injury with loss of consciousness, focal neurological findings on physical examination or when a central nervous system process is suspected from history or examination.

More studies are needed to determine if the risk factors for falls among older workers are similar to older persons in the community. However, until more information is known, an older worker who falls, whether at work or not, deserves a full fall risk factor assessment and appropriate intervention to improve workplace safety and maintain employability.

## Conclusion

The future increase in numbers and age of older persons in the workplace will impact the practice of occupational medicine. To better manage these older workers, occupational physicians may increasingly need to draw upon the principles and experience of geriatric medicine. Mild cognitive impairment and falls in the workplace are two examples of syndromes associated with ageing that can have impact to the occupational health of older workers. Further research into the occupational health problems of older workers is also needed.

## Competing interests

The author(s) declare that they have no competing interests.

## Authors' contributions

GCHK and DK conceived and drafted the manuscript. Both authors read and approved the final manuscript.
